# A Very Fast Image Stitching Algorithm for PET Bottle Caps

**DOI:** 10.3390/jimaging8100275

**Published:** 2022-10-07

**Authors:** Xiao Zhu, Zixiao Liu, Xin Zhang, Tingting Sui, Ming Li

**Affiliations:** School of Electronic Information, Shanghai DianJi University, Shanghai 201306, China

**Keywords:** machine vision, PET bottle cap, camera calibration, image stitching, defect detection

## Abstract

In the beverage, food and drug industry, more and more machine vision systems are being used for the defect detection of Polyethylene Terephthalate (PET) bottle caps. In this paper, in order to address the result of cylindrical distortions that influence the subsequent defect detection in the imaging process, a very fast image stitching algorithm is proposed to generate a panorama planar image of the surface of PET bottle caps. Firstly, the three-dimensional model of the bottle cap is established. Secondly, the relative poses among the four cameras and the bottle cap in the three-dimensional space are calculated to obtain the mapping relationship between three-dimensional points on the side surface of the bottle cap and image pixels taken by the camera. Finally, the side images of the bottle cap are unfolded and stitched to generate a planar image. The experimental results demonstrate that the proposed algorithm unfolds the side images of the bottle cap correctly and very fast. The average unfolding and stitching time for 1.6-megapixel color caps image can reach almost 123.6 ms.

## 1. Introduction

Polyethylene Terephthalate (PET) bottle caps are widely used in the medical, beverage and food industries. During the process of bottle cap production, surface defects such as scratches or deformations are unavoidable. In order to ensure product quality, surface defect detection is very essential. Traditional defect detection methods are mainly based on manual work. Its disadvantages include low efficiency, high working intensity and low accuracy. With the development of computers and image processing algorithms, defect detection methods using machine vision technology instead of human eyes have improved efficiency and accuracy [1,2].

Nevertheless, it is difficult to obtain a whole image of the bottle cap through one camera simultaneously. Therefore, obtaining a complete cylindrical bottle cap image plays an important role in the bottle cap quality inspection process. Several works have applied multiple cameras placed around a bottle cap to capture images of the bottle cap, and defect detection is performed directly on the captured images. However, due to the non-planar surface of the bottle cap, the cylindrical label is distorted and compressed during the projection imaging process. In addition, there is too much of the overlapping area between the captured image in order to obtain a complete and clear side of the bottle cap. The former can affect the results of the inspection, and the latter can increase the computational cost of defect detection. The collected real images of the bottle cap need to be spliced into a two-dimensional plane 360° panoramic view, which can be completed by using image stitching technology [3,4]. Image stitching technology is the registration and fusion of several adjacent images or photos with overlapping areas to form a 360° or wide-view panoramic image.

Many scholars have conducted a lot of work on image stitching [5,6,7]. Image stitching algorithms are basically divided into area-based methods and feature-based methods. Generally, area-based methods establish the transformation relationship between the image to be registered and the reference image by determining similarity measures. The disadvantage of these methods is that, if the transformation amplitude between the images is slightly large, the method can easily be affected, and the speed of registration is very slow. Feature-based methods extract the image features, perform feature matching and calculate the corresponding relationship between the features to find the transformation relationship between the images. Among the feature-based algorithms, the Scale Invariant Feature Transform (SIFT) feature detector [8], Speeded-Up Robust Features (SURF) feature detector [9] and Oriented Fast and Rotated Brief (ORB) feature detector [10] are common. Generally, these methods are relatively stable, fast and have a wide range of adaptation, especially when there are sufficient reliable features in the scene. Liang et al. [11] and Xu et al. [12] used image stitching algorithms based on features for bottle labels. This approach adopted four cameras that surrounded the bottle label to collect the full side images. Subsequently, since the label in the images was not planar, the cylindrical back-projection [13] transform was performed on these images after image preprocessing. Finally, the method based on SIFT was used to stitch the images. Pang et al. [14] applied image stitching technology for the production of “Tai Chi” animation. A handheld mobile phone was used to collect images under the conditions of three fixed points around the object. Then, the cylindrical projection and the stitching algorithm based on features was adopted to the images.

There are also other methods that are different from the traditional image stitching methods above that are applied in different scenarios. Pahwa et al. [15] presented a simple and accurate system to capture images of a given scene using a spirally moving camera system and displayed the panoramic stitched images in unity for an interactive 3D display. In this approach, prior geometrical information in scenes was used to complete the image stitching. Kang et al. [16] proposed a novel image alignment method based on deep learning and iterative optimization to solve the image stitching under low-texture environments, aimed at constructing a cylindrical panorama from a sequence of images. Fu et al. [9] presented a cylindrical image mosaic method based on fast camera calibration in indoor and tunnel scenarios. The key contribution of this work was that a checkerboard calibration board was used to make it in the overlapping field of view of two adjacent images. Then, the images were stitched using registration parameters obtained by calibration. The image mosaic process was less time consuming compared with traditional methods based on image features. Wu et al. [17] proposed a road scene mosaic method using multi-cameras for the application of cross-regional traffic surveillance scenarios. This approach firstly calibrated the multi-camera through their common information. Then, the projection transformation relationship between the two cameras was obtained. The proposed inverse projection idea and translation vector relationship were used to achieve the mosaic of two traffic-monitoring road scenes. Zhang et al. [18] presented a cylindrical label image stitching method with a multi-camera around the label. In this method, the label image was located by cameras, and a mathematical model was built. Then, the adjacent images were stitched together to obtain an unfolding image of the cylindrical label. Although good results for the cylindrical label were achieved, the method could not be directly applied to the bottle caps, and the execution speed needed further improvement.

**Figure 1:**
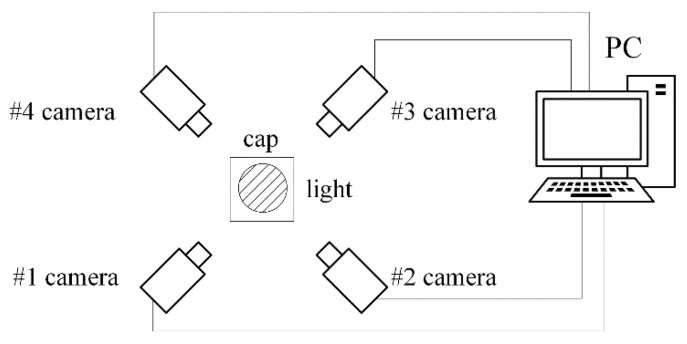
Structure of hardware system.

Therefore, existing stitching models are not suitable for reconstructing the surface of bottle caps or do not meet the requirements for fast and real-time stitching. In the spiral imaging system of our panoramic of bottle cap surfaces, four cameras surround the bottle cap as closely as possible at a 90° interval, as shown in Figure 1. Effective methods should be based on the following observations: (1) A multi-camera is adopted, and the planar assumption of the scene is invalid for us because we collect images of a bottle cap. (2) The bottle cap surface is lacking texture, and the above feature-based method cannot handle the scenes to achieve the image stitching of the bottle cap. Therefore, a Fast Image Stitching Algorithm (FISA) is proposed for PET bottle caps, and the spatial relationship information obtained by camera calibration between cameras and the geometry of the bottle cap is utilized. According to this information, a four-camera coordinate system and a cylinder bottle cap model can be established. In addition, the mapping relationship between the three-dimensional points on the side surface of the bottle cap and the image pixel points are determined. Next, the best view of cameras for the bottle cap need to be solved. The cylindrical back-projection and the interpolation operations are carried out in the regions of the best view of cameras. Finally, the flattened side images are stitched together. The experimental results show that the stitching algorithm can unfold the full side images rapidly and correctly, and the algorithm execution speed meets the real demands. In particular, the contribution of this paper is as follows:

- This paper proposes a FISA model based on projective geometry for PET bottle caps. The method can quickly unfold the full side image of the bottle cap, which can lay the foundation for subsequent defect detection.
- This paper provides several settings with different image quality and different computational times. In actual applications, the settings can be flexibly chosen to meet the actual needs.

## 2. The FISA Algorithm

### 2.1. Algorithm Framework

The structure of the hardware system is shown in Figure 1. The system is mainly composed of four sets of industrial cameras, lenses, LED light sources and one PC. The camera model is Hikvision MV-CA016-10UC (Hangzhou Hikvision Digital Technology Co., Ltd., Hangzhou, China), which is a 1.6-megapixel color camera. The focal length of the lens is 6 mm. Four cameras are mounted surrounding the bottle cap at 90° intervals horizontally, and the side images of the bottle cap are collected and transmitted to the PC.

**Figure 2:**
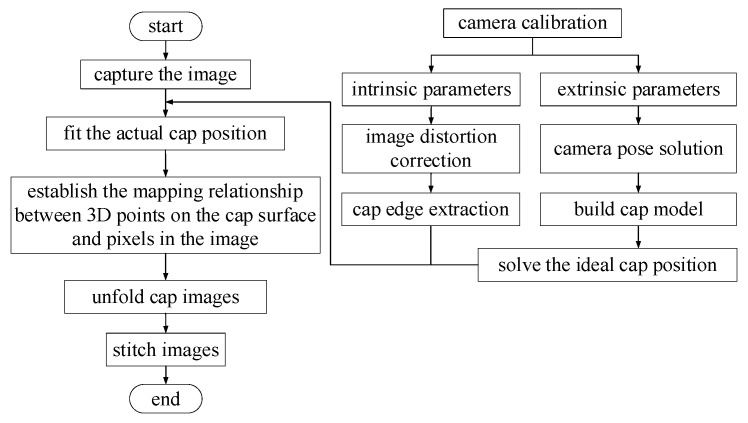
Algorithm flowchart.

The flowchart of the proposed algorithm is shown in Figure 2. Through calibration, the intrinsic and extrinsic parameters of the camera can be obtained. Then, a four-camera coordinate system and the three-dimensional (3D) bottle cap model are built. After that, the mapping relationship between the cap’s 3D points and corresponding image pixels is established. Finally, the cylindrical bottle cap images are projected onto a rectangular plane.

### 2.2. Four-Camera Coordinate System

#### 2.2.1. Geometric Model of the Camera Imaging System

**Figure 3:**
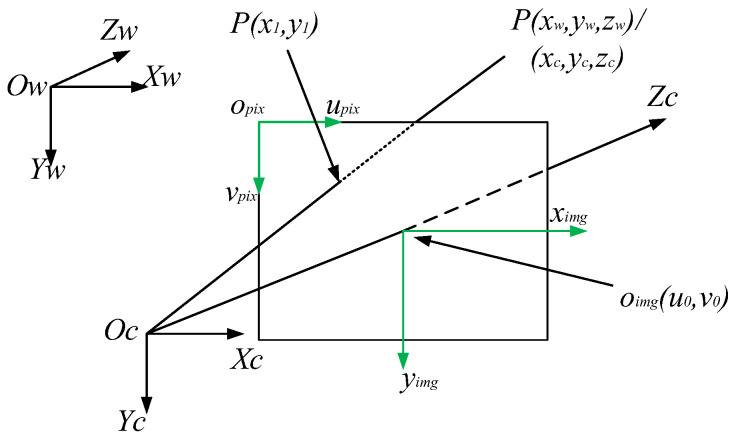
Geometric model of the camera imaging system.

The imaging principle of the camera is the basis of the method in this paper. The geometric model of the camera imaging system [19] is shown in Figure 3. There are four coordinate systems: the world coordinate system $X_{w}Y_{w}Z_{w}$, the camera coordinate system $X_{c}Y_{c}Z_{c}$, the image coordinate system $x_{img}o_{img}y_{img}$ and the pixel coordinate system $u_{pix}o_{pix}v_{pix}$.

When the lights reflected from an object’s surface are converged through the lens to a point (focal point), the object’s image is formed on the imaging plane. For the convenience of observation, the imaging plane is assumed to be located between the pinhole and the object. The imaging direction of the subject is consistent with the actual direction. $o_{c}$ is the origin of the camera coordinate system $X_{c}Y_{c}Z_{c}$. The plane $X_{c}O_{c}Z_{c}$ is parallel to the imaging plane $x_{img}o_{img}y_{img}$. $Z_{c}$ is the optical axis. The distance between the optical center and the imaging plane is the focal length f. Both the image coordinate system $x_{img}o_{img}y_{img}$ and the pixel coordinate system $u_{pix}o_{pix}v_{pix}$ are on the imaging plane. $o_{img}$ is the origin of the image coordinate system, whose value is $\left(u_{0},v_{0}\right)$. The relationship between these coordinate systems is defined as:

(1) $$\left[\begin{array}{c} u\\ v\\ 1 \end{array}\right]=\left[\begin{array}{cccc} \begin{array}{c} \alpha f\\ 0\\ 0 \end{array} & \begin{array}{c} 0\\ \beta f\\ 0 \end{array} & \begin{array}{c} u_{0}\\ v_{0}\\ 1 \end{array} & \begin{array}{c} 0\\ 0\\ 0 \end{array} \end{array}\right]\left[\begin{array}{cc} R & t\\ 0 & 1 \end{array}\right]\left[\begin{array}{c} x_{w}\\ y_{w}\\ z_{w}\\ 1 \end{array}\right]=KM\left[\begin{array}{c} x_{w}\\ y_{w}\\ z_{w}\\ 1 \end{array}\right]$$

where *α* and *β* are the scale factors of the length and pixel value along horizontal and vertical axes, respectively. *R* represents the 3 × 3 rotation matrix, and *t* represents the 3×1 translation vector. *K* is the camera intrinsic parameter matrix, and *M* is the camera extrinsic parameter matrix.

#### 2.2.2. Solving the Four-Camera Coordinate System

Firstly, Zhang’s [20] calibration method is used to calibrate the four cameras respectively, and thus the intrinsic parameters of the four cameras are obtained. The intrinsic parameters are composed of the focal length *f*, the distortion coefficient, main point coordinates $\left(u_{0},v_{0}\right)$, etc., which establish the mapping relationship from the pixel coordinate system to the camera coordinate system. Then, the extrinsic parameters of the four cameras need to be solved. The extrinsic parameter matrix is composed of the rotation matrix *R* and the translation matrix *t*. 

**Figure 4:**
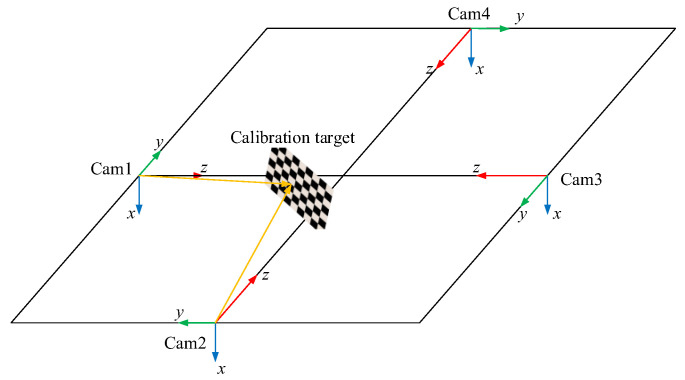
The extrinsic parameter calibration.

As is shown in Figure 1, the four cameras are mounted surrounding the bottle cap at an interval of approximately 90°. In fact, because it is complicated to set the four cameras apart at precise degree intervals, instead we obtain the precise position and pose relationship of each camera by calibrating the extrinsic parameters. In Figure 4, cameras 1 and 2 are used to shoot the same calibration plate.

It is assumed that there is a 3D point on the calibration plate, and the 3D point is expressed as $P(x_{w},y_{w},z_{w})$ in the world coordinate system. The 3D point is projected to a pixel point $p_{1}(u_{1},v_{1})$ in the image of the calibration plate captured by camera 1. The relationship of the 3D point and the pixel point can be expressed by:

(2) $$\left[\begin{array}{c} u_{1}\\ v_{1}\\ 1 \end{array}\right]=K_{1}\left[\begin{array}{cc} R_{1} & t_{1}\\ 0 & 1 \end{array}\right]\left[\begin{array}{c} x_{w}\\ y_{w}\\ z_{w}\\ 1 \end{array}\right]=K_{1}M_{1}\left[\begin{array}{c} x_{w}\\ y_{w}\\ z_{w}\\ 1 \end{array}\right]$$

where $K_{1}$ is the intrinsic parameters of camera 1, $M_{1}$ is the extrinsic parameters and $R_{1}$ and $t_{1}$ refer to the rotation matrix and the translation matrix, respectively, which are both the extrinsic parameters of camera 1.

In the same way, the 3D point is projected to a pixel point $p_{2}(u_{2},v_{2})$ in the image of the calibration plate captured by camera 2. The relationship of the 3D point and the pixel point can be expressed by:

(3) $$\left[\begin{array}{c} u_{2}\\ v_{2}\\ 1 \end{array}\right]=K_{2}\left[\begin{array}{cc} R_{2} & t_{2}\\ 0 & 1 \end{array}\right]\left[\begin{array}{c} x_{w}\\ y_{w}\\ z_{w}\\ 1 \end{array}\right]=K_{2}M_{2}\left[\begin{array}{c} x_{w}\\ y_{w}\\ z_{w}\\ 1 \end{array}\right]$$

where $K_{2}$ is the intrinsic parameters of the camera 2, $M_{2}$ is the extrinsic parameters and $R_{2}$ and $t_{2}$ refer to the rotation matrix and the translation matrix, respectively, which are both the extrinsic parameters of camera 2.

Therefore, $K_{1}$ and $K_{2}$ are known, so the points $\left(x_{c1},y_{c1},z_{c1}\right)$ and $\left(x_{c2},y_{c2},z_{c2}\right)$, for which the 3D point $P(x_{w},y_{w},z_{w})$ is projected on camera coordinate systems of camera 1 and camera 2, can be obtained, respectively. The points $\left(x_{c1},y_{c1},z_{c1}\right)$ and $\left(x_{c2},y_{c2},z_{c2}\right)$ can be written as:

(4) $$\left[\begin{array}{c} x_{c1}\\ y_{c1}\\ z_{c1}\\ 1 \end{array}\right]=\left[\begin{array}{cc} R_{1} & t_{1}\\ 0 & 1 \end{array}\right]\left[\begin{array}{c} x_{w}\\ y_{w}\\ z_{w}\\ 1 \end{array}\right]=M_{1}\left[\begin{array}{c} x_{w}\\ y_{w}\\ z_{w}\\ 1 \end{array}\right]$$

(5) $$\left[\begin{array}{c} x_{c2}\\ y_{c2}\\ z_{c2}\\ 1 \end{array}\right]=\left[\begin{array}{cc} R_{2} & t_{2}\\ 0 & 1 \end{array}\right]\left[\begin{array}{c} x_{w}\\ y_{w}\\ z_{w}\\ 1 \end{array}\right]=M_{2}\left[\begin{array}{c} x_{w}\\ y_{w}\\ z_{w}\\ 1 \end{array}\right]$$

Thus, Equation (6) can be obtained by Equations (4) and(5), which defines the pose relationship between camera 1 and 2.

(6) $$\left[\begin{array}{c} x_{c1}\\ y_{c1}\\ z_{c1}\\ 1 \end{array}\right]=M_{1}M_{2}{}^{-1}\left[\begin{array}{c} x_{c2}\\ y_{c2}\\ z_{c2}\\ 1 \end{array}\right]$$

This is also the rotation and translation relationship between camera 1 and 2. Similarly, the rotation and translation relationship between camera 2 and 3, and even between camera 3 and 4, can be obtained.

Finally, the camera coordinate systems of four cameras are transformed into one coordinate system, where camera 1 serves as the origin (i.e., the four-camera coordinate system).

### 2.3. Building the Cap Model and Solving the Ideal Cap Pose

Firstly, the 3D point cloud of the bottle cap can be expressed by the following Equations (7) and (8).

(7) $$\begin{array}{c} \theta =p_{n}/(\pi \times R/180)\\ p_{n}\in \{1,2,3\cdots N_{pr}\} \end{array},$$

(8) $$\{\begin{array}{c} x=R\cos\theta \\ y=R\sin\theta \\ z=s\\ s\in \{1,2,3\cdots H\} \end{array},$$

Here, *R* and *H* are the radius and height of the bottle cap, respectively. $N_{pr}$ means the number of pixels in each row after the cap side image is unfolded, which equals the perimeter of the cap. $p_{n}$ represents the arc length of the cap surface along the horizontal direction. θ is the degree of the central angle of the circle corresponding to the length of the arc $p_{n}$.

**Figure 5:**
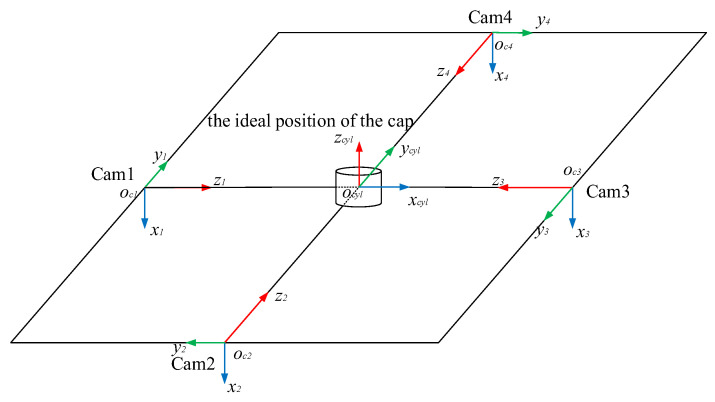
The ideal position and pose of the bottle cap.

The next step is to solve the ideal pose of the cap. As shown in Figure 5, there are four camera coordinate systems: $x_{1}y_{1}z_{1}$, $x_{2}y_{2}z_{2}$, $x_{3}y_{3}z_{3}$ and $x_{4}y_{4}z_{4}$. In the four-camera coordinate system, the coordinate origins of the four coordinate systems of cameras are $o_{c1}(0,0,0)$, $o_{c2}(x_{o2},y_{o2},z_{o2})$, $o_{c3}(x_{o3},y_{o3},z_{o3})$ and $o_{c4}(x_{o4},y_{o4},z_{o4})$, respectively.

The 3D point $o_{cyl}(x_{m},y_{m},z_{m})$ is obtained from Equation (9), which means the center of the four-camera coordinate system.

(9) $$\{\begin{array}{c} x_{m}=\frac{0+x_{o2}+x_{o3}+x_{o4}}{4}\\ y_{m}=\frac{0+y_{o2}+y_{o3}+y_{o4}}{4}\\ z_{m}=\frac{0+z_{o2}+z_{o3}+z_{o4}}{4} \end{array}$$

The direction cosines of the space vector $\vec{o_{c1}o_{cyl}}(x_{m},y_{m},z_{m})$ in the $x_{1}$, $y_{1}$ and $z_{1}$ directions are cosδ, cosη and cosγ, respectively, which can be obtained by:

(10) $$\{\begin{array}{c} \cos\delta =\frac{x_{m}}{\sqrt{x_{m}{}^{2}+y_{m}{}^{2}+z_{m}{}^{2}}}\\ \cos\eta =\frac{y_{m}}{\sqrt{x_{m}{}^{2}+y_{m}{}^{2}+z_{m}{}^{2}}}\\ \cos\gamma =\frac{z_{m}}{\sqrt{x_{m}{}^{2}+y_{m}{}^{2}+z_{m}{}^{2}}} \end{array}$$

The corresponding direction angles are δ, η and γ, which represent the *X*-axis direction of the ideal bottle cap model’s pose. Next, a plane is constructed where the point $o_{cyl}(x_{m},y_{m},z_{m})$ passes through and where the origins of the four camera coordinate systems are closest. In addition, the normal line of the plane is the *Z*-axis direction of the ideal cap model’s pose. Next, the cross product of the *X*-axis and *Z*-axis is the *Y*-axis direction of the ideal cap model’s pose. Thus, the ideal pose of the cap is obtained. This coordinate system is defined as the bottle cap coordinate system. Finally, five coordinate systems are established, including cameras 1, 2, 3, 4 and the bottle cap coordinate system.

### 2.4. Extracting the Bottle Cap Edge

In order to obtain the actual pose information of the bottle cap in a 3D space, it is necessary to solve the relationship between the ideal and actual poses of the bottle cap. In this paper, the edge information of the cap image is extracted first. Then, the solved ideal pose of the cap is used to fit the cap edge in the image to determine the actual position of the cap and this part is in the next subsection.

**Figure 6:**
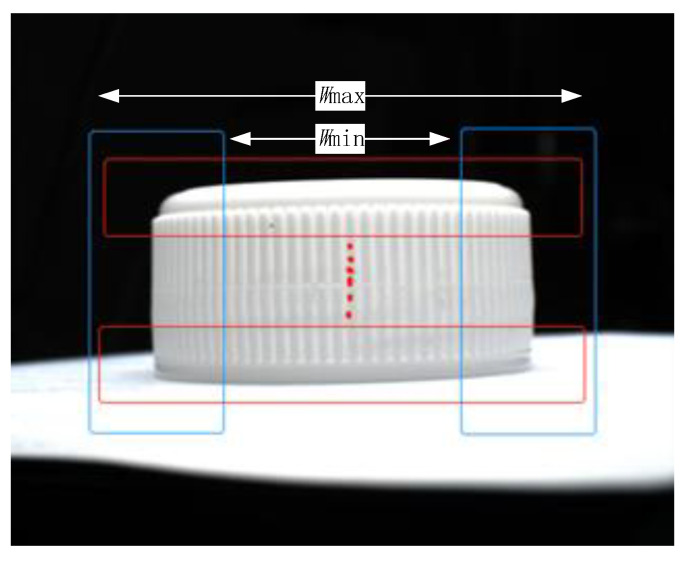
Edge extraction process.

The details of the edge extraction are as follows. Firstly, distortion correction is applied to the image. Then, the pixel coordinates of the edge of the bottle cap are obtained by using edge detection algorithms such as Canny or Marr–Hildreth [21]. In order to improve the efficiency of edge extraction, the Canny edge detection algorithm combined with a fuzzy rule is used. This allows one to define a fuzzy membership function [22], which describes the features of good edges. The advantage of this approach is its flexibility to deal with extra edges. This approach can flexibly restrict the range of edge extraction (the blue rectangle in Figure 6) by the fuzzy membership function:

(11) $$f(x)=\{\begin{array}{ll} \frac{x-w_{\min}}{a}+1 & w_{\min}-a\leq x<w_{\min}\\ 1 & w_{\min}\leq x\leq w_{\max}\\ \frac{w_{\max}-x}{a}+1 & w_{\max}<x\leq w_{\max}+a\\ 0 & \begin{array}{c} \begin{array}{l} x<w_{\min}-a\;or\\ w_{\max}+a<x \end{array} \end{array} \end{array}$$

where $\left[w_{\min},w_{\max}\right]$ represents the range of edge extraction, $\left[w_{\min}-a,w_{\min}\right)$ and $\left(w_{\max},w_{\max}+a\right]$ represent the flexible (i.e., fuzzy) range and a is set to 10. 

Moreover, a sliding window (the red rectangle in Figure 6) is applied to extract a straight edge perpendicular to the red rectangle.

### 2.5. Fitting the Actual Cap Pose

**Figure 7:**
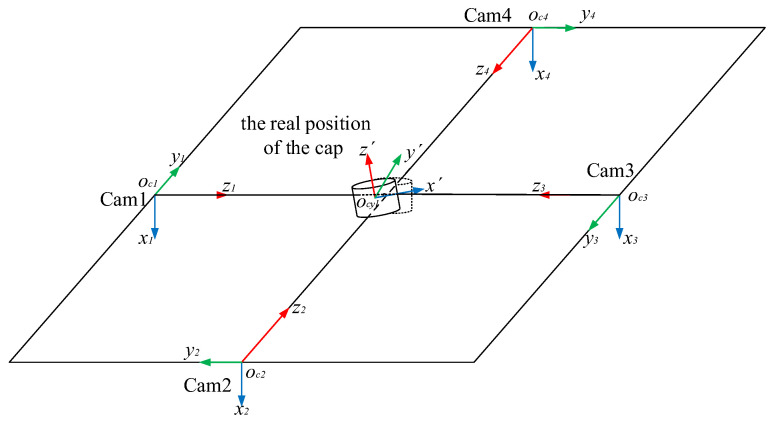
The actual pose of the cap.

Next, the extracted edge information and the solved ideal pose of the cap are used to fit the actual pose of the cap, as shown in Figure 7. The pixel coordinates of the edge points $A_{i}$ are transformed to the camera coordinate system of camera 1 by:

(12) $$\left[\begin{array}{c} u_{1}\\ v_{1}\\ 1 \end{array}\right]=K_{1}\left[\begin{array}{c} x_{c1}\\ y_{c1}\\ z_{c1}\\ 1 \end{array}\right]$$

Finally, the coordinate of camera 1 and the edge feature points $A_{i}$ extracted from the image of the bottle cap are transformed to the cap coordinate system xyz again to obtain the space vector $\vec{o_{c1}A_{i}}$. The distance of the space vector $\vec{o_{c1}A_{i}}$ and $\vec{o_{cyl}z}$ is (the distance of the skew line) $d_{1i}$, which is the distance from the spindle of the ideal bottle cap to the edge of the actual bottle cap. Similarly, for cameras 2, 3 and 4, the distances $d_{2i}$, $d_{3i}$ and $d_{4i}$ can be calculated, respectively. 

The error E can be obtained by subtracting the actual bottle cap radius R from the distances (i.e.,$d_{1i}$, $d_{2i}$, $d_{3i}$ and $d_{4i}$):

(13) $$E=\sum_{j=1}^{4}\sum_{i=1}^{n}(d_{ji}-R{)}^{2}$$

**Figure 8:**
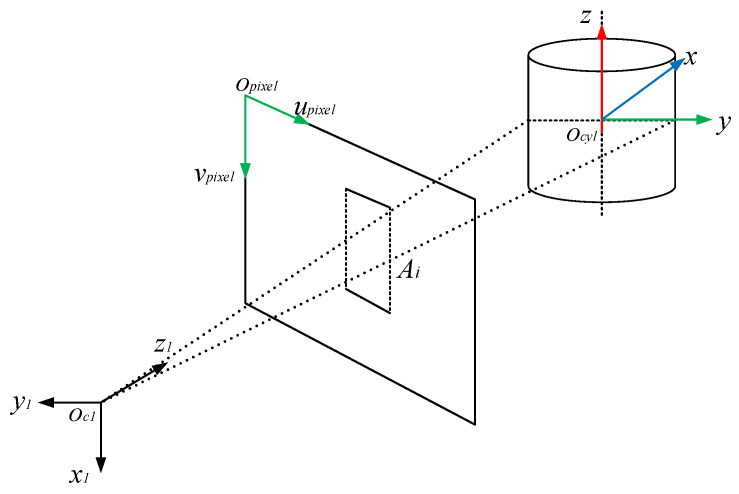
Imaging model of the bottle cap edges.

The least squares method is used to minimize the error value E; therefore, the principal axis $o_{cyl}{}^{\prime }z^{\prime }$ of the actual cap is obtained, as shown in Figure 8. Then, the perpendicular line from the coordinate of camera 1 to the principal axis $o_{cyl}{}^{\prime }z^{\prime }$ is drawn to obtain the *X*-axis of the actual cap position. The cross product of the *X*-axis and the principal axis $o_{cyl}{}^{\prime }z^{\prime }$ is the *Y*-axis of the actual cap position. So far, the pose of the actual bottle cap has been solved. 

### 2.6. Determining the Best View of Cameras for the Bottle Cap

**Figure 9:**
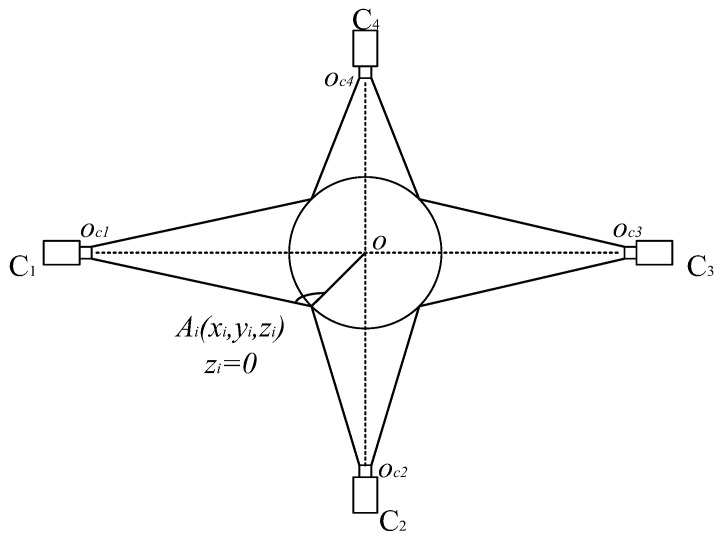
The best view of cameras for the bottle cap.

In this section, it is important to solve the best view of cameras for the bottle cap to determine which regions of the cap are seen best from which camera. An observation angle $∠OA_{i}O_{c1}$ is able to determine the best view of cameras, as shown in Figure 9. It can be seen that, when the observation angle is larger, the camera’s view for the cap is smaller, and the observation regions of cameras for the cap are smaller. This method allows the observation regions of each camera to be stitched together without overlaps and intervals. 

Equation (15) is used to solve the best observation angle for each camera. The specific details of the process are as follows. In the bottle cap coordinate system, the 3D coordinates of the origin $o_{c1}$ of camera 1 are subtracted by the 3D points $A_{i}(x_{i},y_{i},z_{i})$ on the cap surface (to reduce the calculation, let $z_{i}=0$) to obtain the vector $\vec{o_{c1}A_{i}}{=(a}_{x}{,b}_{y}{,c}_{z})$. The direction cosines of the vector $\vec{o_{c1}A_{i}}$ in the *x*, *y* and *z* directions are $\frac{a_{x}}{\left|\vec{o_{c1}A_{i}}\right|}$, $\frac{b_{y}}{\left|\vec{o_{c1}A_{i}}\right|}$ and $\frac{c_{z}}{\left|\vec{o_{c1}A_{i}}\right|}$, respectively. Then, the 3D coordinate points $A_{i}(x_{i},y_{i},z_{i})$ of the cap surface are multiplied by the direction cosine of the corresponding direction of the vector $\vec{o_{c1}A_{i}}$ and are summed together to obtain the observation value $\beta_{1}$ of camera 1, as shown in Equation (14). The corresponding observation angle of the observation value is $∠OA_{i}O_{c1}$, as shown in Figure 9.

(14) $$\beta_{1}=x_{i}*\frac{a_{x}}{\left|\vec{o_{c1}A_{i}}\right|}+y_{i}*\frac{b_{y}}{\left|\vec{o_{c1}A_{i}}\right|}+z_{i}*\frac{c_{z}}{\left|\vec{o_{c1}A_{i}}\right|}$$

The observation values of the four cameras $\beta_{1}$, $\beta_{2}$, $\beta_{3}$ and $\beta_{4}$ are calculated, respectively. The four values are compared, and when $\beta_{j}$ is the largest, its corresponding observation angle is the best observation range of camera *j*.

(15) $$\{\begin{array}{c} \vec{o_{cj}A_{i}}{=(a}_{x}{,b}_{y}{,c}_{z}),\;(j=1,2,3,4)\\ \beta_{j}=\max(x_{i}*\frac{a_{x}}{\left|\vec{o_{cj}A_{i}}\right|}+y_{i}*\frac{b_{y}}{\left|\vec{o_{cj}A_{i}}\right|}+z_{i}*\frac{c_{z}}{\left|\vec{o_{cj}A_{i}}\right|}),\;(j=1,2,3,4) \end{array}$$

### 2.7. Image Unfolding and Stitching

**Figure 10:**
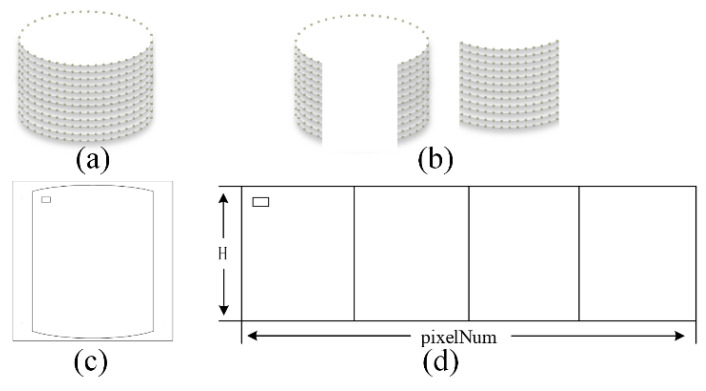
The unfolding and stitching flowchart of the cap: (**a**) The 3d model of the cap; (**b**) The best observation regions of the camera; (**c**) The cylindrical back-projection on the best observation regions of the camera; (**d**) The stitched result of the (**c**).

**Figure 11:**
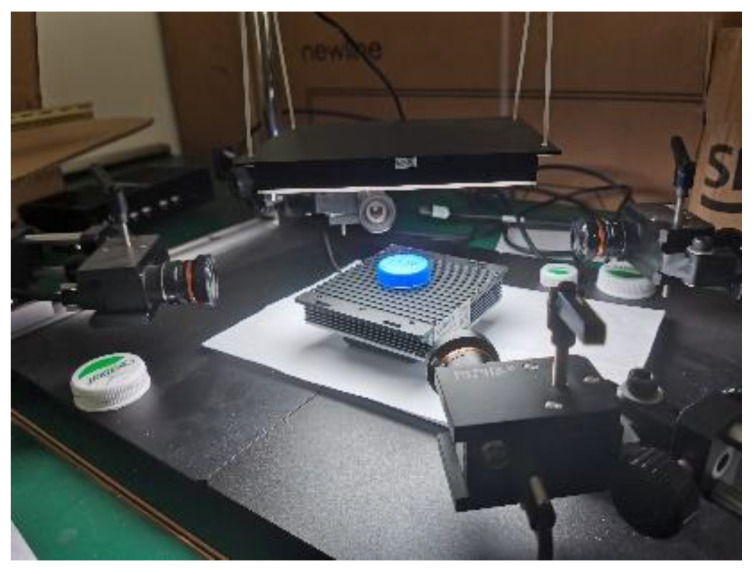
Structure of experimental system.

The calculated 3D points of the best view of bottle cap areas (Figure 10b) are cylindrical back-projected on the rectangular plane (Figure 10c) and are stitched to generate a full unfolding image of the cap side (Figure 10d), as shown in Figure 11. Image fusion techniques can be used to overcome the shortcomings of an unnatural appearance after image stitching. They include the weighted fusion technique, pyramid fusion technique, gradient domain fusion technique, etc. [23]. In this paper, the simple fading-in and fading-out fusion algorithm is chosen to fuse the images.

To summarize, the steps of the new stitching strategy for cylindrical bottle cap surfaces can be given as follows:
**The procedure of stitching images of bottle cap sides**.**Step 1:** The intrinsic parameters of the four cameras are calibrated by Zhang’s calibration method, respectively. Then, the extrinsic parameters of the four cameras are calibrated by the approach designed in this paper. Finally, a four-camera coordinate system is established. **Step 2:** The center of the four-camera coordinate system is found, and a new coordinate that represents the ideal position of the cap in the four-camera coordinate system is established with the center as the origin. **Step 3:** A 3D point cloud model of the cap with this new coordinate origin as its center is established. **Step 4:** A set of images of the cap side is captured by the four-camera system.
**Step 5:** Edge feature extraction is performed for bottle cap side images after image preprocessing. **Step 6:** The actual position of the cap is determined by exploiting the ideal position and the edge feature information of the cap. **Step 7:** The best view of cameras for the bottle cap is solved to determine which regions of the cap are seen best from which camera. The best observation regions of cameras for the cap can be obtained.
**Step 8:** According to the best observation regions of cameras, the images belonging to the regions (i.e., region of interest) are cylindrical back-projected and are stitched to generate a full unwrapping image of the cap side.

## 3. Experiments

In order to evaluate the performance of our proposed method, we implemented the algorithms proposed in this paper. The test machine used in our experiments was equipped with an Intel(R) Core (TM) i5-9300H CPU at 2.40 GHz (with four cores and eight threads), an NVIDIA GeForce GTX 1660ti GPU and 6 GB of physical memory. The operating system for our test machine was Windows 10. The experimental system is shown in Figure 11. All four cameras were firstly calibrated to obtain the intrinsic and extrinsic parameters to use to rectify images and to build the four cameras’ spatial coordinates. The images were acquired from different angles of the cap side.

**Figure 12:**
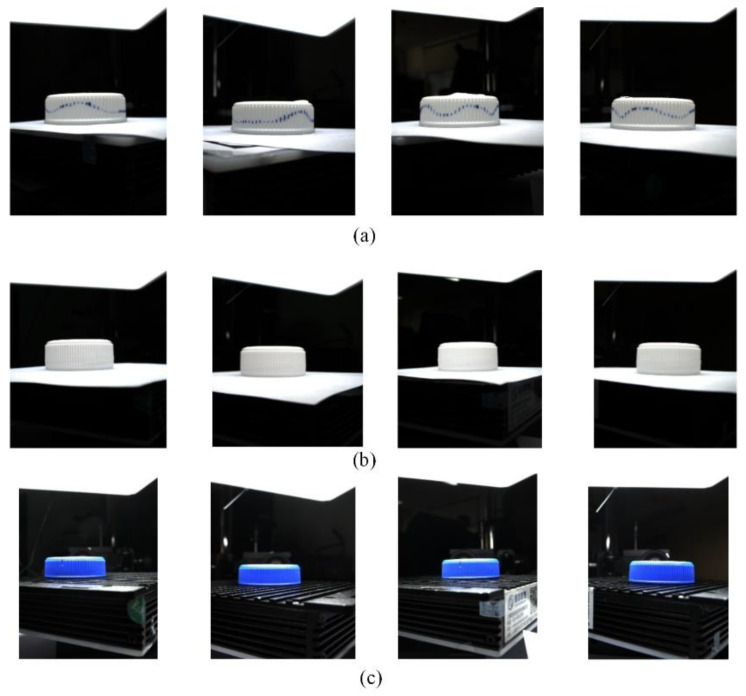
(**a**) Sample 1; (**b**) Sample 2; (**c**) Sample 3.

In order to prove the universality of the proposed algorithm, several different kinds of caps were used in the experiments, and three of them are shown in Figure 12.

### 3.1. Results Analysis

The ideal spatial pose of the cap should be in the center of the four-camera coordinate system; however, the actual spatial pose of the cap may have deviated. Therefore, we utilized the edge feature information of the cap images to determine the actual spatial pose of the cap. A good edge extraction result helped locate the actual bottle cap pose more accurately.

**Figure 13:**
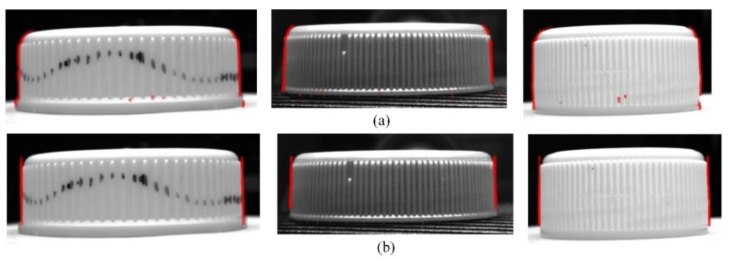
Edge detection results: (**a**) Edge detection results of samples 1, 2 and 3 using our methods; (**b**) Edge detection results of samples 1, 2 and 3 only using the Canny edge detection algorithm.

In addition to the Canny edge detection algorithm used for edge feature extracting, a sliding window and a fuzzy rule were used to extract the straight edge and to restrict the range of edge extraction. The effect of this can be seen in the bottom of Figure 13. The top of Figure 13 shows the effect of only the Canny edge detection algorithm being used. There are some outliers, and the extracted edges are not straight on it.

#### 3.1.1. The Unfolding Images of the Caps

**Figure 14:**
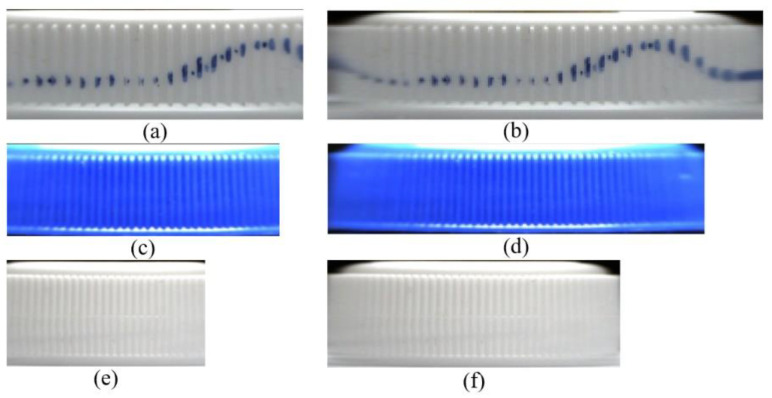
Results after the cylindrical back-projection: (**a**,**c**,**e**) Results after the cylindrical back-projection on the best observation regions of cameras of the three samples; (**b**,**d**,**f**) Results after the cylindrical back-projection on the image of the full side of the caps of the three samples.

**Figure 15:**
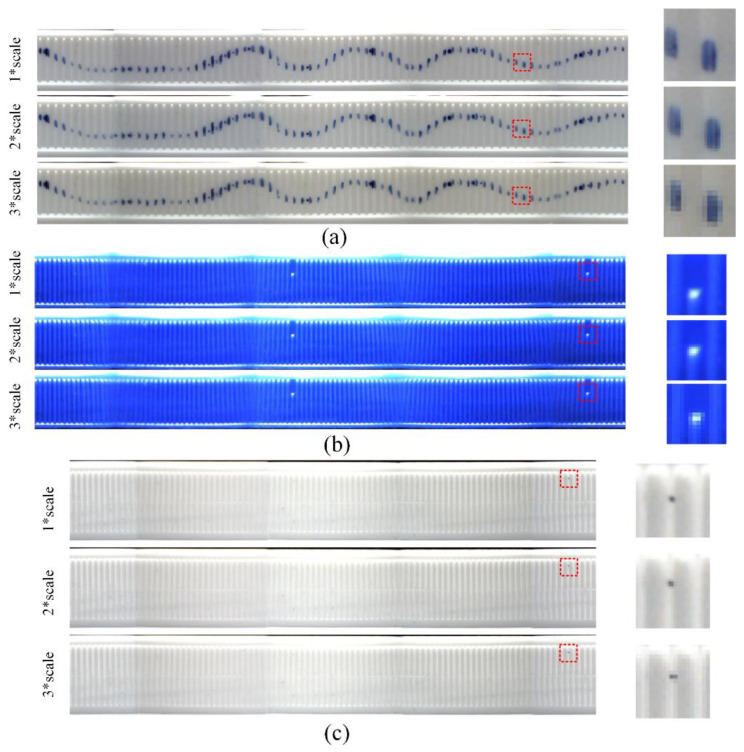
The stitching results: (**a**) The stitching results of the images of the sample 1; (**b**) The stitching results of the images of the sample 2; (**c**) The stitching results of the images of the sample 3.

Since the four-camera coordinate system and the cylindrical coordinate system of the actual bottle cap were established, the mapping relationship between the spatial 3D points of the cap surface and the pixel points of the cap’s images could be obtained. Next, the images of the bottle caps belonging to the best observation regions of cameras were used with the cylindrical back-projection to generate flattened images of the caps, as shown in the left of Figure 14. In the process, since we did not perform the cylindrical back-projection on the image of the side of the full caps [11,19], as shown in the right of Figure 14, this could reduce the computation cost significantly. Finally, the flattened images of the caps were stitched to generate a full unfolding image of the bottle cap, and the effects of the three samples are shown in Figure 15.

In addition, in order to explore the relationship between image quality and computation cost, we conducted a set of experiments that projected the images of bottle caps onto rectangular planes with several settings, including performing projection transformation with a pixel area, two times the pixel area and three times the pixel area (equivalent to performing downsampling in the projection transformation). The settings were denoted by a 1 × scale, 2 × scale and 3 × scale, respectively. It can be seen clearly in Figure 15.

We utilized a blue marker to draw a continuous curve on the bottle cap side in sample 1 to test the effect of the stitching. As shown in Figure 15a, the curves properly coincide with each other in the stitched result image. It also can be seen that the bottom part of the cap is a little larger than the radius of the main part of the cap (the middle part), so there is a little error in the splicing of the bottom part of the cap. Moreover, as illustrated in Figure 15b, the vertical texture of the joint in sample 3’s stitching result is slightly inclined. This is because sample 3’s cap is a frustum cone-like cap rather than a normal cylinder, resulting in minor joint defects. However, these had almost no impact on the subsequent defect detection of the bottle caps.

#### 3.1.2. Application

Cap defect detection results: Existing image segmentation methods are mainly divided into the following categories: threshold-based, edge-based [24,25] and methods based on specific theories. Since the captured image usually contains spot-like Gaussian noises and may have uneven surfaces and inhomogeneous illuminations, the contrast between the defects and the background information is usually not that high. If the threshold segmentation is performed directly in the spatial domain, it results in the incomplete extraction of defect information or even error extraction. Therefore, Gaussian filtering was firstly used in this paper to suppress the image background noises. Then, the Sobel-based algorithm was adopted [26]. The advantages of the Sobel operator include good anti-noise and small calculations. After Sobel edge detection, the contrast between the bottle cap defect and the background of the neighboring domain increased. Finally, the precise detection and location of bottle cap defects could be completed with morphological processing and feature extraction operations.

**Figure 16:**
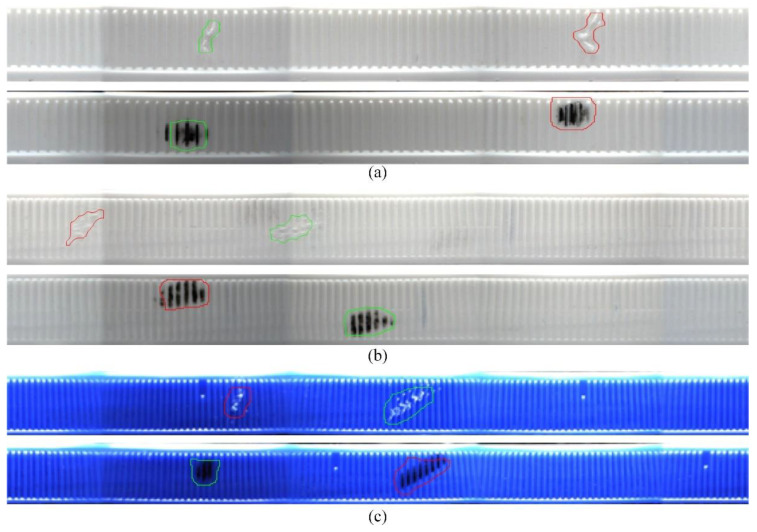
The defect detection results for three stitched sample images using FISA algorithm: (**a**) The defect detection results of scratch and oil stain defects on sample 1; (**b**) The defect detection results of scratch and oil stain on sample 2; (**c**) The defect detection results of scratch and oil stain on sample 3.

Sobel edge detection, morphological processing and feature extraction methods were used to detect defects such as scratches and oil stains in the stitched image, and the effects of the three samples are shown in Figure 16.

### 3.2. Performance Analysis

In order to evaluate the stitching speed of bottle cap images at different image qualities, experiments were conducted 100 times on the three samples in this paper, respectively. The average unfolding and stitching time for different samples on different scales is shown in Table 1. It can be seen from Table 1 that the times for the proposed algorithm were 172.2, 159.5 and 123.6 ms on the 1× scale. As a comparison, the time spent on the 2× scale and 3× scale conditions was greatly reduced, and the corresponding image quality was also reduced. The time required to complete the unfolding and stitching was reduced by almost 53% on the 3× scale compared to the 1× scale. In actual applications, we can flexibly choose the settings to meet the actual needs.

The method in Ref. [19] is the latest stitching method used for cylindrical labels currently, so it is included for comparison. It can be seen from Table 2 that the actual execution times that used the stitching method in Ref. [19] were 237.2, 220.6 and 171.4 ms. In other words, by using our algorithm, the time required to complete the unfolding and stitching was reduced by almost 40%.

Overall, we used the known geometric information, including the camera pose relationship obtained by camera calibration and the cylindrical bottle cap model. The mapping relationship between the 3D points on the bottle cap surface and the camera imaging to 2D planar pixel points was established without time-consuming feature point searching and matching, which are usually used in traditional stitching methods based on features. 

In fact, the most time-consuming part of this process was likelly fitting the actual bottle cap pose, if the cap edge was not extracted accurately. The other parts were matrix operations, which were similar to the operations of other stitching methods after obtaining the transformation matrix. In addition, the images of the bottle caps belonging to the best observation regions of cameras, rather than images of the sides of the full caps, were used with the cylindrical back-projection to generate flattened images of the caps, and this could also reduce the computation cost significantly.

Finally, defect detection was performed on the stitched side images of the bottle cap. Defect detections were performed 100 times for each sample. The average detection time for the three samples was 7.74 ms, 7.28 ms and 6.97 ms, respectively, as shown in Table 3.

## 4. Conclusions

This paper proposes a stitching method for the images of bottle caps, in which the surfaces of a bottle cap are reconstructed to generate an unwrapped plane image of the bottle cap’s sides. Firstly, in the image stitching method, the four-camera coordinate system is established through calibration, and the cylindrical bottle cap model is solved. Then, the position and pose relationship between the four cameras and the bottle cap is established in a 3D space to obtain the mapping relationship between the 3D points of the bottle cap and the pixels of the image taken by the camera. Next, the best view of the cameras for the bottle caps needs to be solved. The unfolding and interpolation are only carried out in the regions of the best view of the cameras. Finally, the pixels of the bottle cap image are rearranged to form a complete side image of the bottle cap, resulting in a good imaging effect and fast executing speed.

In order to evaluate the performance of the proposed approach in terms of the unfolding speed of the bottle caps, several experiments were conducted on three samples of the bottle caps. The experimental results show that, for the bottle cap images captured by a 1.6-megapixel color camera, the fastest average unfolding and stitching time was about 61.6 ms on the 3× scale. In addition, several settings with different image quality and computational time are provided. In actual applications, the settings can be flexibly chosen to meet the actual needs. In addition, tubes with different radii will be our future work.

## Figures and Tables

**Table 1:** The average unfolding and stitching time $t_{m}$ for 1.6-megapixel color cap images on different scales.

Sample	Scale	Resolution	Radius (mm)	Height (mm)	$t_{m}$ Time (ms)
Sample 1	1 × scale	1440 × 1080	24.05	16.3	172.2
Sample 2	1 × scale	1440 × 1080	20.2	19.4	159.5
Sample 3	1 × scale	1440 × 1080	20.1	12.5	123.6
Sample 1	2 × scale	1440 × 1080	24.05	16.3	101.3
Sample 2	2 × scale	1440 × 1080	20.2	19.4	92.7
Sample 3	2 × scale	1440 × 1080	20.1	12.5	74.4
Sample 1	3 × scale	1440 × 1080	24.05	16.3	82.7
Sample 2	3 × scale	1440 × 1080	20.2	19.4	75.4
Sample 3	3 × scale	1440 × 1080	20.1	12.5	61.6

**Table 2:** The average unfolding and stitching time $t_{m}$ for 1.6-megapixel color cap images.

Sample	Resolution	Radius (mm)	Height (mm)	$t_{m}$ Time (ms)
Sample 1	1440 × 1080	24.05	16.3	172.2
Sample 2	1440 × 1080	20.2	19.4	159.5
Sample 3	1440 × 1080	20.1	12.5	123.6
Sample 1 [19]	1440 × 1080	24.05	16.3	237.2
Sample 2 [19]	1440 × 1080	20.2	19.4	220.6
Sample 3 [19]	1440 × 1080	20.1	12.5	171.4

**Table 3:** The average defect detection time for the stitched images.

Sample	The Image Size after Stitching	The Average Defect Detection Time (ms)
Sample 1	554 × 65	7.74
Sample 2	554 × 53	7.28
Sample 3	554 × 46	6.97

## Data Availability

Not applicable.
